# Accidental ingestion of an endodontic file: A case report and literary review

**DOI:** 10.1016/j.radcr.2022.09.071

**Published:** 2022-10-21

**Authors:** Ariana R. Tagliaferri, Gabriel Melki, Crystal Feghali, Yana Cavanagh

**Affiliations:** Department of Gastroenterology, St. Joseph's University Medical Center, Paterson, NJ 07470, USA

**Keywords:** Accidental ingestion, Endodontic file, Dental procedure, Endoscopy, Endoscopic retrieval, Gastroenterology

## Abstract

Ingestion and aspiration can be accidental or intentional events in both adults and children. Approximately 1500 people in the United States die from ingestion of foreign bodies annually. Patients with cognitive disabilities, neurological disorders, elderly age or incarcerated patients carry the highest risk of intentional and/or accidental ingestion of foreign objects. Although uncommon, ingestion of foreign objects during dental procedures can be potentially life-threatening and increased awareness is important. Sharp objects ingested from dental procedures can cause impaction, obstruction, hemorrhage, or perforation and may need endoscopic or surgical intervention. Herein we report a case of a 22-year-old male, who underwent routine dental cleaning and accidentally ingested an endodontic file, retrieved from the ascending colon endoscopically without complications.

## Introduction

Ingestion and aspiration of foreign bodies have been well documented [1], [2], [3]. In general, ingestion is more common than aspiration, although occurs more frequently in the pediatric population [1,2]. Usually incidents of ingestion or aspiration occur in children younger than 3 years old with a male to female ratio of 1.2:1 [1]. Between accidental and intentional incidents, approximately 1,500 children and adults die each year following ingestion of foreign bodies in the United States [1]. Patients with cognitive disabilities or motor deficits, such as those who are elderly, physically disabled or mentally challenged have a higher risk of ingestion and aspiration events [1,3]. Additionally, patients taking opiates, anti-depressants or other sedatives, alcohol abusers, and patients with neurological disorders such as those with Parkinson's disease, dementia, epilepsy or strokes are also at an increased risk [1].

After ingestion there is a 12:1 chance that the object will pass over the respiratory tract and enter the digestive tract [2]. A retrospective, cross-sectional study identified variables that affected visualization of foreign bodies after ingestion and demonstrated that of 168 endoscopies, 52.4% of objects could be retrieved from the mouth at time of presentation [4]. Of these, elderly patients were more likely to have the foreign bodies removed from the mouth, and objects that were visible on imaging were visualized and retrieved endoscopically [4]. As such, the majority of foreign objects can be managed non-surgically, however up to 1% may require surgical interventions [2]. The majority of ingested foreign objects include boluses of food, bones, seeds, beans, batteries and coins [1,4]. It is a rare, but documented and serious adverse event for accidental ingestion of dental instruments during dental procedures [2,3]. Although the true incidence of this is unknown, previous studies have estimated the incidence to be from 0.00012% to 0.004% [2]. Many dental instruments and appliances have been documented, such as dental burs, endodontic files, rubber dam clamps, dental mirrors, implant instruments, and barbed broaches [3]. Although it is rare, complications can be serious and possibly fatal and thus awareness of these adverse events and how to manage them are crucial.

Herein we report a case of a 22-year-old male, who underwent routine dental cleaning and accidentally ingested an endodontic file, retrieved from the ascending colon endoscopically without complications.

## Case report

**Fig. 1:**
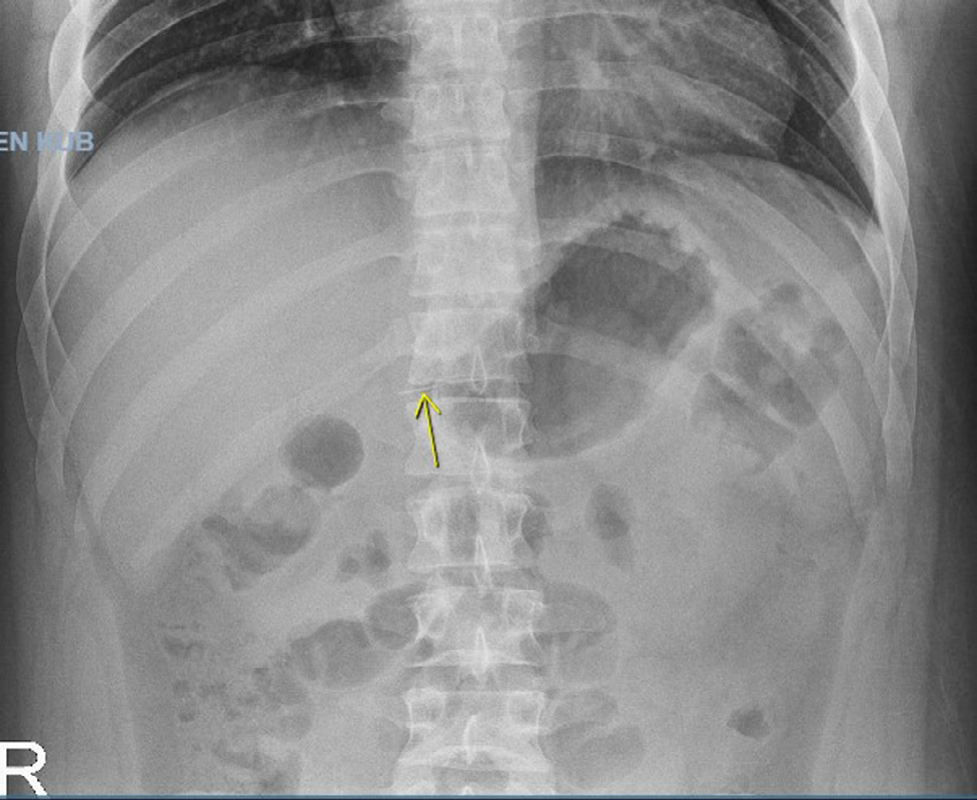
X-ray of abdomen demonstrating foreign object in the gastric antrum. Initial X-ray of the kidneys, ureters and bladder revealed a 4 mm linear radiopaque foreign body overlying the midline of the upper abdomen. Arrow indicates foreign body likely in the gastric antrum. No free air or obstruction identified.

**Fig. 2:**
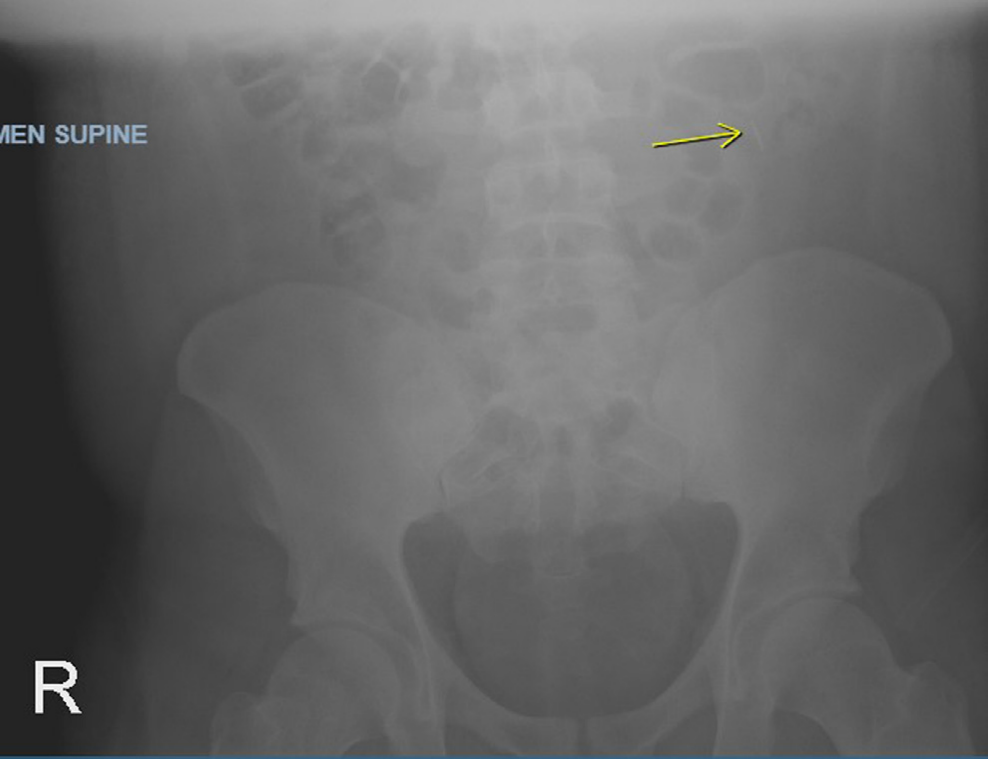
X-ray of abdomen demonstrating foreign object in left mid-abdomen. A repeat X-ray of the kidneys, ureters and bladder revealed a linear radiopaque foreign body overlying bowel loops on the left side of the mid-abdomen (arrow). The exact location could not be identified. No free air noted on film.

**Fig. 3:**
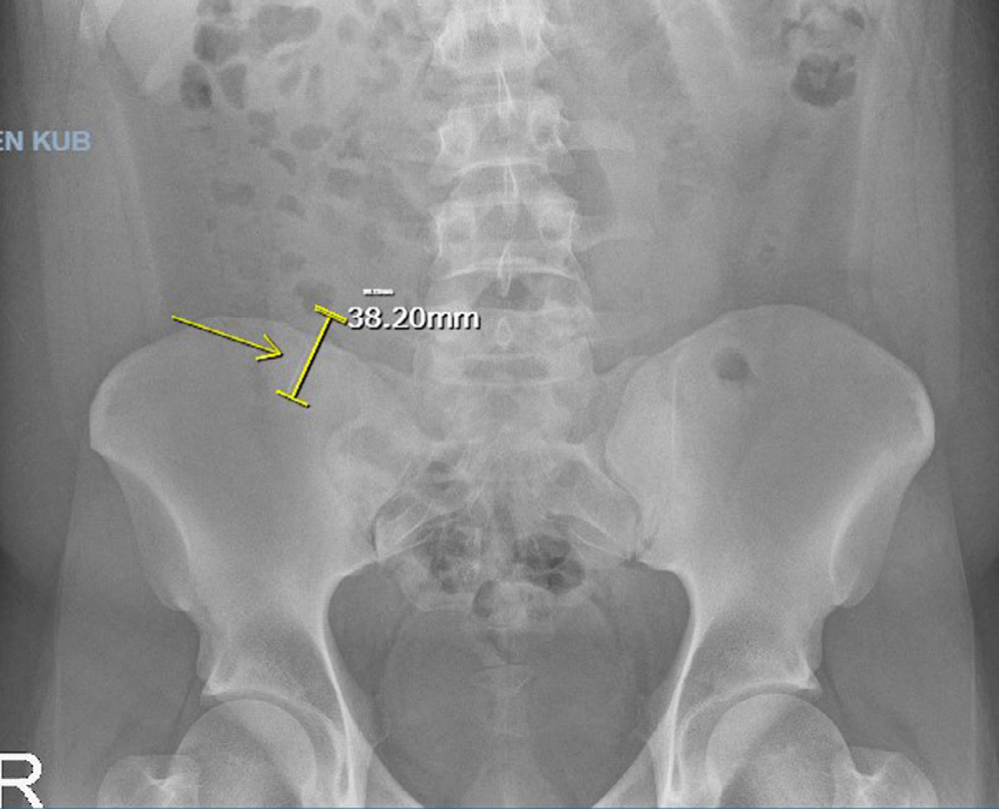
X-ray of abdomen demonstrating partial small bowel obstruction. A repeat X-ray of the kidneys, ureters and bladder revealed mildly dilated small bowel loops representative of ileus or partial small bowel obstruction. Arrow indicates a radiopaque foreign body in the right lower abdomen without evidence of mass effect. There is no evidence of free air. Measurement approximately 38 mm.

**Fig. 4:**
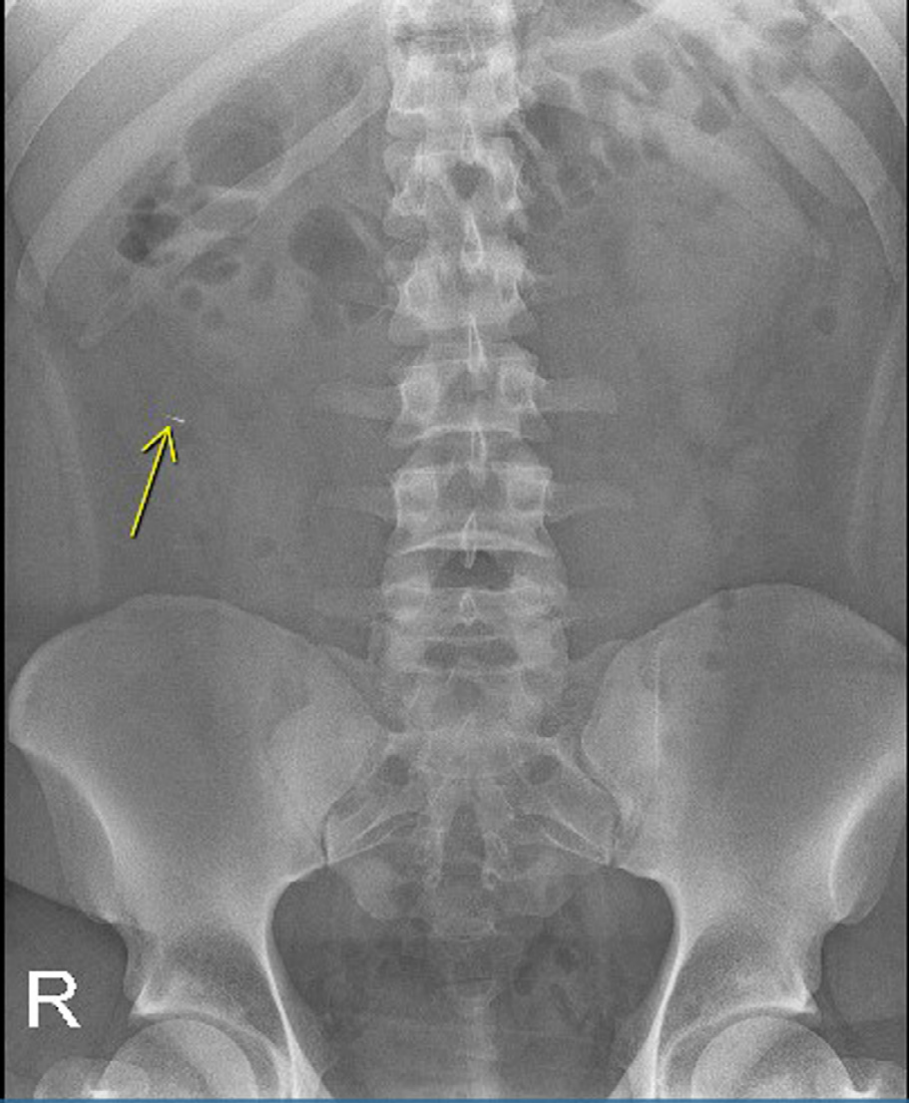
X-ray of abdomen demonstrating migrating foreign object. A repeat X-ray of the kidneys, ureters and bladder revealed redemonstration of a linear radiopaque foreign body in the right mid-abdomen, migrating likely into the ascending colon (arrow).

**Fig. 5:**
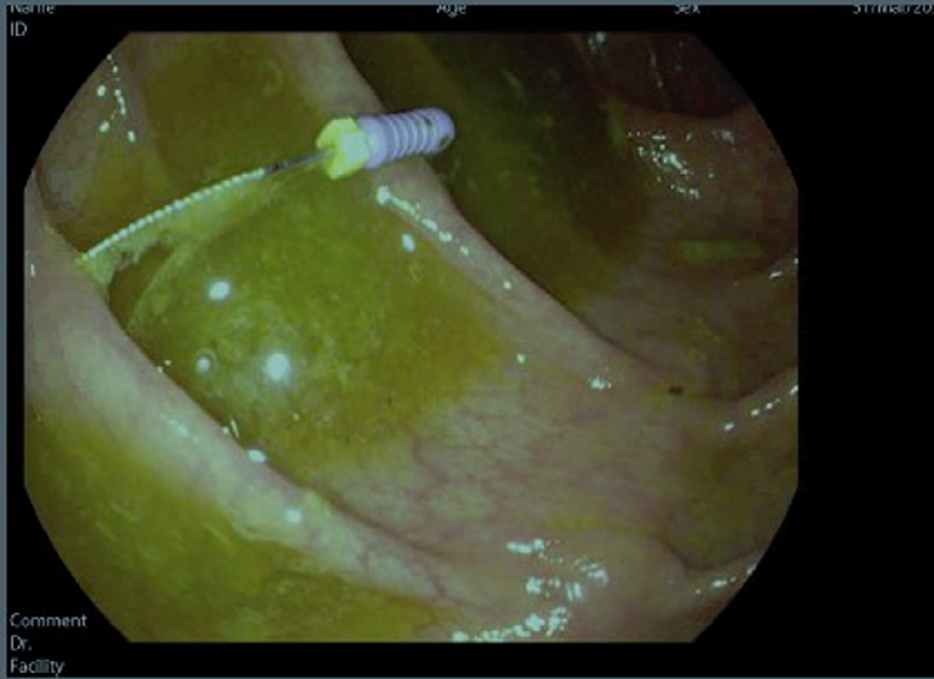
Colonoscopy images from the ascending colon. The colonic mucosa was normal. Arrow indicates a foreign body was found in the ascending colon. Removal was accomplished using a Roth net and rat root forceps. This was extracted into the rectum and manually removed.

**Fig. 6:**
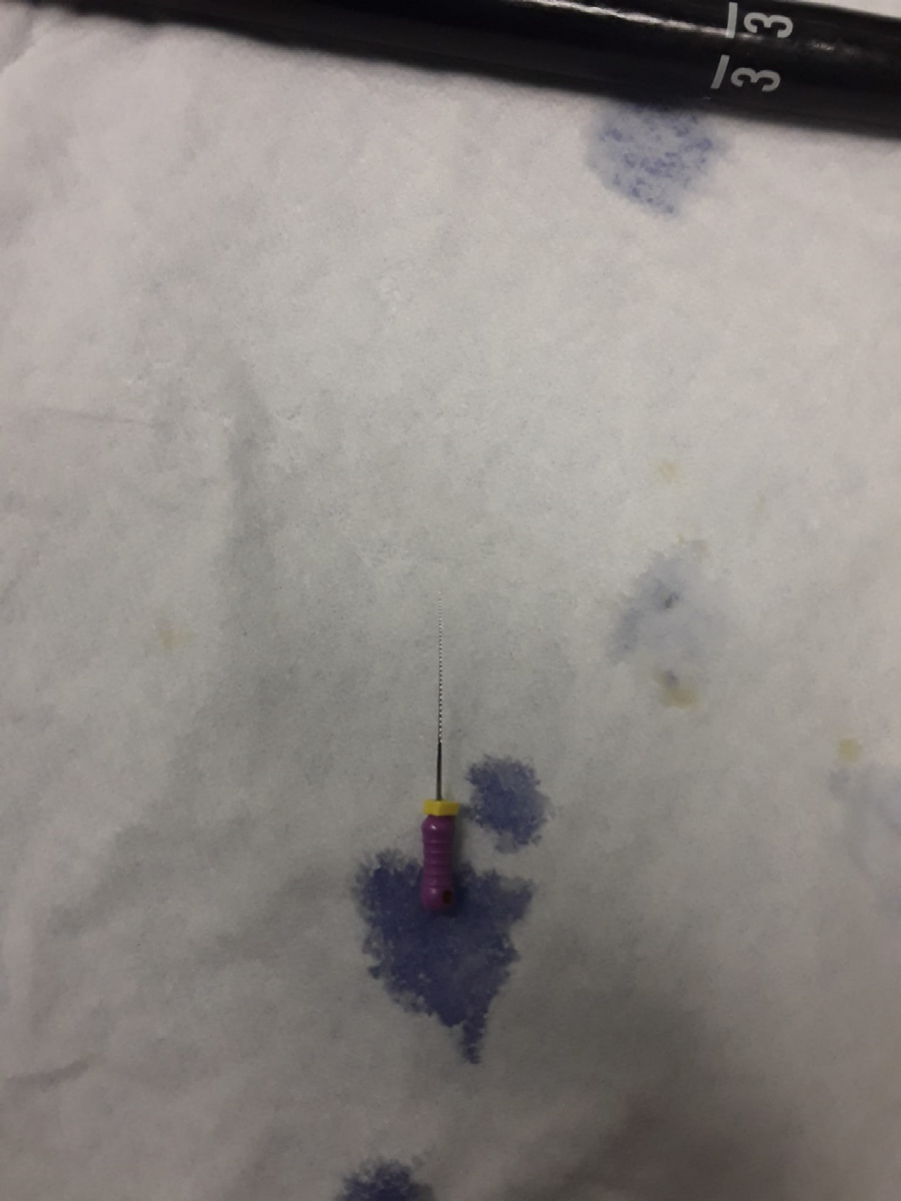
Dental file after extraction from the rectum. The dental file after removal from the rectum.

A 22-year-old male with past medical history of obesity presented to the Emergency Department (ED) after accidently swallowing a dental file during a procedure. The patient reported minimal epigastric pain and he denied nausea, vomiting, fever or chills. He was able to swallow his saliva without difficulty, denied hoarseness and was not in respiratory distress. On physical exam he was afebrile and normotensive, saturating 99% on ambient air. He had normoactive bowel sounds and had mild epigastric tenderness. Laboratory studies including a comprehensive metabolic panel, complete blood count and lipase were within normal limits. X - ray films of the neck, chest and abdomen were performed revealing a 4 mm linear radiopaque foreign body identified overlying the midline of the upper abdomen likely in the gastric antrum (Fig. 1). Since the sharp end of the dental file was in the gastric antrum, a decision was made to pursue an enteroscopy for foreign body retrieval. The enteroscopy revealed a normal esophagus, gastric, duodenal and jejunal mucosa, and the foreign body was not found. The patient was kept in the hospital overnight for serial abdominal exams and repeat imaging approximately 6 hours following the procedure. The X-ray showed a previously identified linear radiopaque foreign body now overlying bowel loops in the left mid-abdomen (Fig. 2). The exact location however could not be determined from this image alone. The following morning the patient reported he was no longer in abdominal pain and physical exam did not reveal tenderness. He had repeat imaging revealing a radiopaque foreign body in the right lower abdomen (Figs. 3 and 4). The patient was started on a clear liquid diet, given a bowel prep and underwent a colonoscopy that afternoon. The foreign body was retrieved from the ascending colon. The sharp end was piercing the mucosa, superficially. The removal of the dental file was accomplished using a Roth net and removed with rat tooth forceps. This was extracted into the rectum and was manually removed to minimize trauma in the anal canal (Figs. 5 and 6). The patient tolerated the procedure well with no complaints or complications.

## Discussion

Although accidental ingestion is more common in children overall, the second most common cause of accidental ingestion in adults is from dental procedures [1]. In a span of 10 years, 36 or more incidents have been reported [1]. A PubMed database search from 1983 to 2016 revealed that smaller objects, such as archwire fragments or brackets comprised the majority of cases, while only 4 accidents involved larger objects such as a fractured Twin block appliance, intact quadhelix or 3 cm-long Kobayashi ligature [1]. Other common items which have been reported in current literature include second molar buccal tubes, trans-palatal arches, impression materials, toothpicks, files, burs, clamps, removable prostheses, retainers, dental implant screw drivers, mirror heads and extracted teeth [1]. In certain patients with neurological diseases, the incidence may be higher due to reduced gag reflex and decreased metabolism of sedation [3]. Patients who intentionally ingest foreign objects primarily ingest metallic items, and are often prisoners or psychologically disabled persons [5]. Comparatively, accidental ingestion of food or dental objects is common in other demographics [5]. A retrospective analysis demonstrated that metallic items are usually located in the stomach in 64% of cases, whereas food, bones or dental objects are found in the esophagus in 87.5% of cases [5]. This study determined that those who intentionally ingest foreign bodies had a longer duration of impaction and thus had less success with endoscopic or surgical retrieval [5]. Early identification and intervention are imperative in this population [5].

Most items pass spontaneously within 7-10 days; however, management and possible complications are contingent on the size and shape of the object [2,4]. Life threatening complications include impaction, ulceration of the mucosa, obstruction, abscess formation, hemorrhaging or fistula formation [1,^2^,5]. Foreign objects less than 5 cm are likely to pass without complications; however, the pylorus, appendix, sigmoid colon and anal canal are high-risk sites for impaction and perforation [1]. Sharp objects, such as endodontic files have a higher risk of causing these complications, as they pass the curves of the intestines [2,^4^,5]. Moreover, patients with a history of adhesions, hernias, diverticula or inflammatory bowel disease have a higher risk of these complications due to their anatomy [2].

Early imaging can identify the best means of intervention. Objects such as razor blades, batteries, metallic objects, bones and dental equipment are more likely to be retrieved endoscopically or surgically [2,5]. If a foreign body is visualized in the small bowel, double-balloon enteroscopy is the gold standard [2]. Those with objects less than 60 millimeters in length and 25 millimeters in diameters are very low risk for perforation or obstruction and thus can be managed conservatively [2].

Preventative measures include use of a physical barrier such as a rubber dam, adjustment of chair positioning, attachment of tools to floss or a string, or use of a magnetic clip retractor [2,3]. A retrospective study in New Zealand illustrated use of physical barriers in only 44% of dental procedures [2]. Although uncommon, ingestion of foreign objects during dental procedures can be potentially life-threatening and increased awareness is important.

## Conclusion

Ingestion of foreign objects is a rare but potentially life-threatening event that can be minimized through preventative measures during dental procedures.

## Author contributions

Tagliaferri, A.R., wrote the manuscript and performed the literary review. Melki, G., Feghali, C., and Cavanagh. Y assisted with the collection of the patient's data and reviewed and edited the final manuscript. All authors consent to this publication.

## Patient consent

Consent was obtained for the purpose of this paper.
